# Occlusal contact and clearance of posterior implant-supported single crowns designed by two different methods: a self-controlled study

**DOI:** 10.1186/s12903-023-02847-w

**Published:** 2023-03-15

**Authors:** Mingzhu He, Tingting Pu, Qian Ding, Yao Sun, Pengfei Wang, Yuchun Sun, Lei Zhang

**Affiliations:** 1 Department of Prosthodontics, Peking University School and Hospital of Stomatology & National Center of Stomatology & National Clinical Research Center for Oral Diseases & National Engineering Laboratory for Digital and Material Technology of Stomatology & Beijing Key Laboratory of Digital Stomatology & Research Center of Engineering and Technology for Computerized Dentistry Ministry of Health & NMPA Key Laboratory for Dental Materials, 22 South Street，Zhong Guan Cun, 100081, Beijing, Haidian District China; 2 School/Hospital of Stomatology, Lanzhou University, Lanzhou, China; 3 Denture Processing Center, Peking University School and Hospital of Stomatology & National Center of Stomatology & National Clinical Research Center for Oral Diseases & National Engineering Laboratory for Digital and Material Technology of Stomatology & Beijing Key Laboratory of Digital Stomatology & Research Center of Engineering and Technology for Computerized Dentistry Ministry of Health & NMPA Key Laboratory for Dental Materials, Beijing, China; 4 Department of Prosthodontics, The Third Clinic of Peking University School and Hospital of Stomatology, Beijing, China; 5 Center of Digital Dentistry, Peking University School and Hospital of Stomatology & National Center of Stomatology & National Clinical Research Center for Oral Diseases & National Engineering Laboratory for Digital and Material Technology of Stomatology & Beijing Key Laboratory of Digital Stomatology & Research Center of Engineering and Technology for Computerized Dentistry Ministry of Health & NMPA Key Laboratory for Dental Materials, Beijing, China

**Keywords:** Occlusal clearance, Implant-supported single crown, CAD/CAM, Accuracy

## Abstract

**Background:**

Precise occlusal design of implant-supported fixed prostheses is difficult to achieve by the conventional wax-up method, often requiring chairside adjustments. The computer-aided design (CAD) method is promising. This study aims to compare the occlusal contacts and clearance of posterior implant-supported single crowns designed by the CAD and conventional methods.

**Methods:**

Sample size calculation indicated fourteen samples per group. Two sets of type-IV plaster casts with a single implant analog inserted in the posterior teeth region were mounted as master casts in a mechanical articulator in maximal intercuspal position (MIP). Seven working cast sets were obtained from each master cast by a closed tray technique, and mounted in MIP. Two implant-supported single crowns were designed with an occlusal clearance to achieve light occlusal contact in each working cast set by CAD and conventional method, separately. For the CAD group, the crown was designed in digital models obtained by scanning the working casts. For the conventional group, wax-up of the crown was prepared on the working casts and scanned to generate a STL file. In the working and master casts, mean and minimum occlusal clearances in the designed occlusal contact area of the both finished prostheses were calculated using the occlusal clearance (OC) and occlusal record (OR) method. The prostheses’ occlusion was evaluated in master casts.

**Results:**

For the evaluation in the working casts, both design methods had similar mean occlusal clearances by the OC method (195.4 ± 43.8 vs. 179.8 ± 41.8 μm; *P* = 0.300), while CAD group resulted in a significantly larger minimum occlusal clearance in the designed occlusal contact area (139.5 ± 52.3 vs. 99.8 ± 43.8 μm; *P* = 0.043). Both design methods had similar mean and minimum occlusal clearances by the OR method (*P* > 0.05). For the evaluation in the master casts, both design techniques had similar mean and minimum occlusal clearances, number and distribution of occlusal contacts, and lateral interference ratios (*P* > 0.05).

**Conclusion:**

Occlusal contact and clearance of posterior implant-supported single crowns designed by the CAD method can be at least as good as those designed by the conventional wax-up method.

## Background

Osseointegrated implants react biomechanically to occlusal force in a manner distinct from natural teeth because they lack the periodontal ligament and have a high threshold of tactile perception [1, 2]. Consequently, dental implants are prone to occlusal overload, which is considered a cause of mechanical complications such as screw loosening and fracture, prosthesis fracture, or even implant fracture, eventually compromising the implant longevity [3, 4]. Therefore, appropriate design of occlusion could affect the implant-supported fixed prostheses longevity. Currently, computer-aided design (CAD) and conventional wax-up are the most commonly used methods to design fixed prostheses, achieving similar occlusion in full crowns [5] and fixed partial dentures [6]. Zhang et al. designed full crowns based on the occlusal surfaces of the pre-preparation teeth, and found a less lateral interference compared with those designed using the library method [7]. Yeliz et al. concluded that full crowns designed by a reference tool were personalized and suitable in occlusion [8]. Therefore, a reference tool can be regarded as an appropriate method to replicate the contralateral tooth morphology when designing an implant-supported single crown.

Occlusion strategy studies indicated that a occlusal clearance of about 10–30 μm on the fixed implant-support single crowns in maximal intercuspal position (MIP) is desired [9–11]. Due to the light occlusal contact of the implant-support single crown, precise occlusal clearance design is difficult to achieve by the conventional wax-up method, often requiring chairside adjustments. However, few studies focused on the occlusal design of implant-supported prostheses using the CAD method. And the effect of occlusal clearance design in implant-supported single crowns using the CAD method remains unclear and worth exploring.

A CAD method to design the occlusion of implant-supported single crowns is presented in this study. The method includes using a reference tool to copy the morphology of the corresponding tooth on the contralateral side to the missing tooth and designing an occlusal clearance using the antagonist tool in a virtual articulator. This study aimed to compare the occlusal contacts and clearance of posterior implant-supported single crowns designed by the CAD and conventional methods. The null hypothesis was that no differences would be found in occlusal contact and clearance outcomes between crowns designed with these two methods on master casts.

## Methods

### The master cast preparation

Two sets of type IV plaster casts from two partially-edentulous patients were selected as master casts for this study. The Biomedical Institutional Review Board of Peking University School of Stomatology approved of using patients’ casts to simulate virtual patients in this study (PKUSSIRB-202,055,068). Inclusion criteria were type IV plaster casts classified as Kennedy class III, a single bone level implant analog (RC, Straumann, Basel, Switzerland) inserted in the posterior teeth region needed restoration, had an even and stable intercuspal occlusion, and the contralateral tooth was in a normal position and had no defects. Exclusion criteria were casts with severe tooth wear and less than three occlusal contact points in MIP [12]. Written informed consent was obtained from the participants prior to their casts’ inclusion in the study.

The two included master cast sets were mounted manually on a mechanical articulator (PROTARevo 7; KaVo Dental GmbH, Biberach, Germany) in MIP with low-expansion gypsum (ZERO-arti; Dentona AG, Dortmund, Germany) by M.H. A 3-kg weight was placed on the articulator. The physical occlusal contacts were determined by a 12-µm metallic polyester film (Arti-Fol metallic Shimstock-film; Dr. Jean Bausch GmbH, Cologne, Germany).

### The working cast preparation

This study was designed as a self-controlled in vitro study. The sample size calculation was based on the results of a preliminary test. Twelve implant-supported single crowns were required per group to maintain a significance level of 0.025 and power of 80% to detect a difference of 20-µm mean occlusal clearance in the master casts, with a common standard deviation of 27.7 μm. In order to avoid insufficient sample size caused by errors in the process of prosthesis manufacturing, 14 samples were included in each group in this study.

**Fig. 1:**
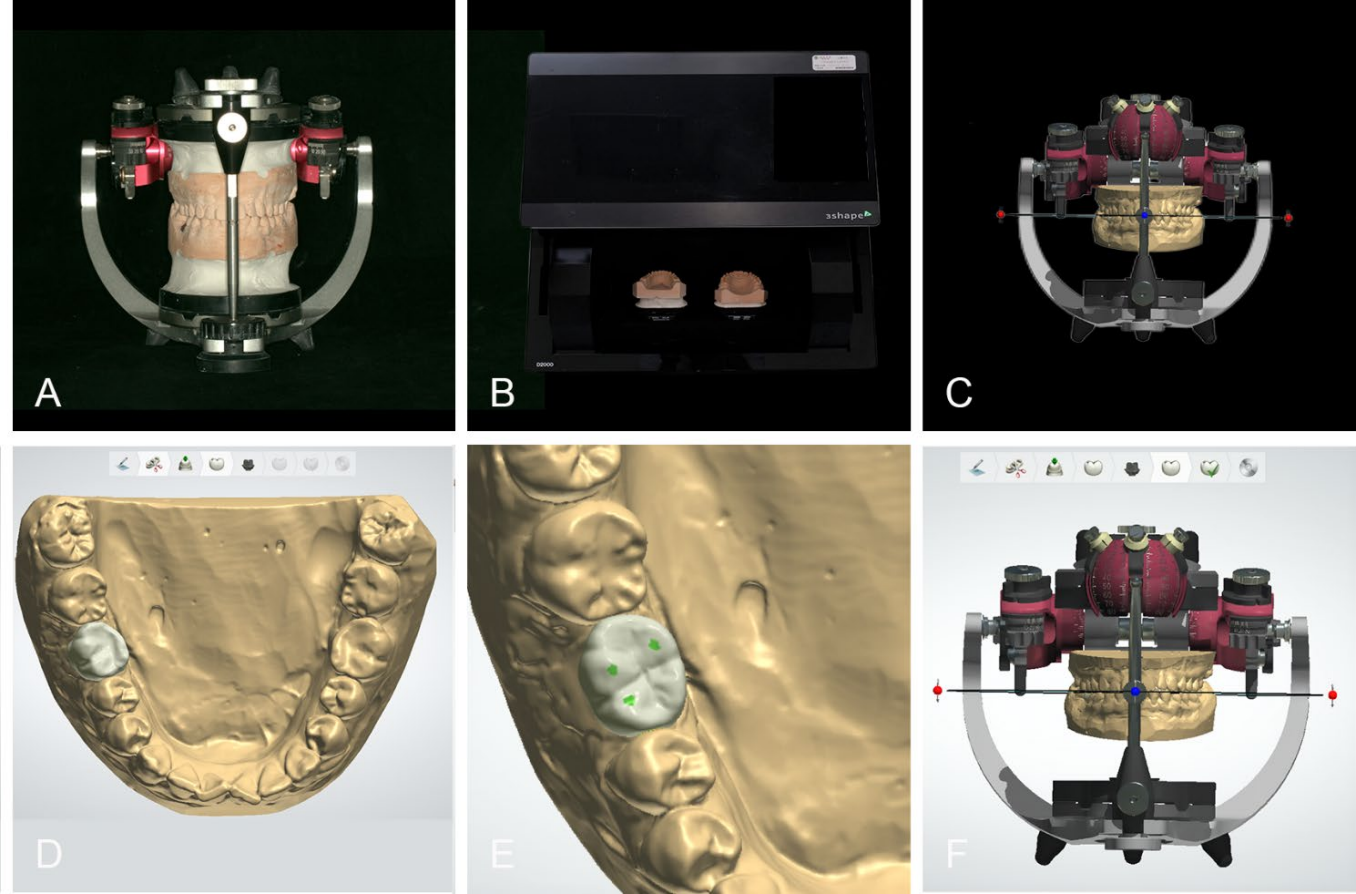
Model scanning procedure and prostheses designed by CAD method: A, mounting casts on mechanical articulator; B, scanning casts with transfer plate; C, mounting casts on virtual articulator; D, reference tool used to copy morphology of contralateral tooth to generate prosthesis; E, “antagonist tool” was used to achieve light and stable cusp-fossa occlusal contact of implant prosthesis; F, virtual articulator helped adjust eccentric occlusion of crowns programmed with mean values of condylar guidance

Closed-tray impressions were performed on the two mounted master cast sets with vinyl polysiloxane (Variotime Light Flow and Dynamix Monophase, Heraeus Kulzer GmbH, Hanau, Germany). Seven replicas of each master cast, 14 working cast sets in total, were made with type IV gypsum stone (Die-stone, Heraeus Kulzer GmbH, Hanau, Germany). Casts with defects or voids were discarded. The working casts were manually mounted on the same mechanical articulator in MIP with low-expansion gypsum by the same researcher M.H. (Fig. 1A). The mounted working casts were stored with a 3-kg weight placed on the articulator. The occlusal contact of all working casts was verified with the 12-µm Shimstock-film. The casts were remounted if an occlusal deviation was found.

### Design of implant-supported single crowns

Two implant-supported single crowns for each set of working casts were designed by the researcher M.H, one using the conventional method and the other using the CAD method in 3Shape Dental System (version 18.1.0, 3Shape A/S, Copenhagen, Denmark).

#### The CAD method

In the CAD group, an occlusal transfer calibration object was mounted on the mechanical articulator and scanned with the transfer plates in a laboratory scanner (D2000 scanner, 3Shape A/S, Copenhagen, Denmark) to calibrate the virtual articulator in 3Shape Dental System. Then the mechanical articulator information was transferred to the virtual articulator [13]. Subsequently, the mounted working casts were scanned with the same transfer plates and automatically set in MIP in the virtual articulator (Fig. 1B,C). A scannable abutment (Scanbody; Straumann, Basel, Switzerland) was used to digitize the position and angulation of the implant at the same time.

In 3Shape Dental System, a reference tool copied the morphology of the contralateral tooth to generate the prosthesis (Fig. 1D), and the occlusion was designed to achieve a stable cusp-fossa contact with the opposing teeth. Based on a clinical pre-test, 80-µm occlusal clearance was considered appropriate for designing an implant-support single crown, requiring minimal clinical occlusal adjustment. Therefore, the occlusal clearance was set to 80 μm using the antagonist tool (Fig. 1E). The virtual articulator adjusted the crowns’ eccentric occlusion with the same occlusal clearance as the centric occlusion, programmed with mean condylar guidance values (protrusive condylar inclination, 35º; Bennett angle, 15º; Fig. 1F) [14]. Then the Standard Triangle Language (STL) file of the prosthesis was generated.

#### The conventional method

A wax-up of implant-supported single crown was made on the working casts mounted on the mechanical articulator. The 80-µm occlusal clearance setting was estimated by extracting two sheets of 38-µm articulating papers (SHOFU, Japan) with light resistance in MIP. The occlusal contact points were indicated with a 100-µm articulating paper (BK 52 Red; Dr. Jean Bausch GmbH, Cologne, Germany). The eccentric occlusion was adjusted on the mechanical articulator with the same condylar guidance settings as in the CAD group. The wax-up was scanned after powdering with Arti-Spray (BK 285, Dr. Jean Bausch GmbH, Cologne, Germany) to obtain a STL file of the designed prosthesis.

### Manufacturing of prostheses

Data of the two crowns were sent to a 5-axis milling machine (Zenotec T1; Wieland Dental Technik GmbH & Co. KG, Pforzheim, Germany). Intrinsically colored monolithic zirconia blocks (Ideal Zirconia; Organical CAD/CAM GmbH, Berlin, Germany.) were milled and sintered following the manufacturer’s recommendations. The sintered crowns were lightly powdered and scanned in a laboratory scanner before coloring and glazing, and the data were saved as STL files. The sintered and corresponding designed crowns were compared by “3D compare” in a three-dimensional (3D) data processing software (Geomagic Control 2014; 3D Systems, North Carolina, USA) to calculate the deviations of prosthesis fabrication. Subsequently, the crown’s axial surface was colored and glazed. Only pits and fissures in the crown’s occlusal surface were colored, while other areas were highly polished.

### Determination of designed occlusal contact area

**Fig. 2:**
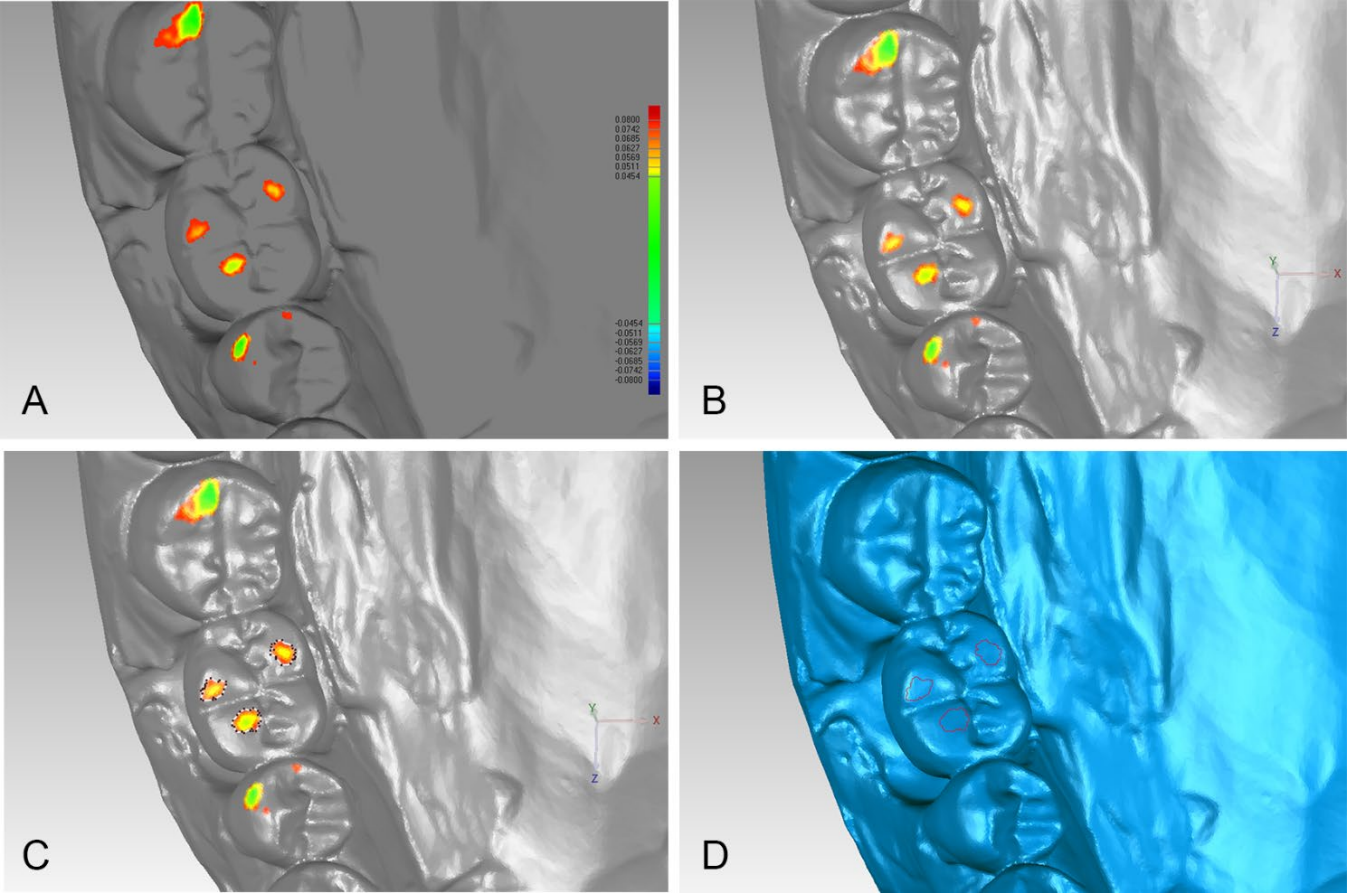
Determination of occlusal contact areas: A, “3D compare” results in designed crown; B, converts result object to polygon object; C, selecting boundaries of designed occlusal contact areas; D, boundaries of occlusal contact areas on crown were determined

The STL files of both crown design methods were imported to the Geomagic software program. The designed occlusal contact areas were identified by “3D compare”, setting the threshold value to 80 μm (Fig. 2). The actual mean occlusal clearance in designed occlusal contact area of the two designed crowns was calculated by “3D compare”.

### Working cast evaluation

The finished crown was temporarily bonded on the abutment (Variobase, Straumann, Basel, Switzerland) in the working casts. The occlusal clearance between the crown and the opposite teeth was qualitatively evaluated by pulling out articulating papers with thicknesses of 12, 30 (Yamahachi Dental, Japan), 100, and 200 μm (BK 52 Red; Dr. Jean Bausch GmbH, Cologne, Germany) in succession.

**Fig. 3:**
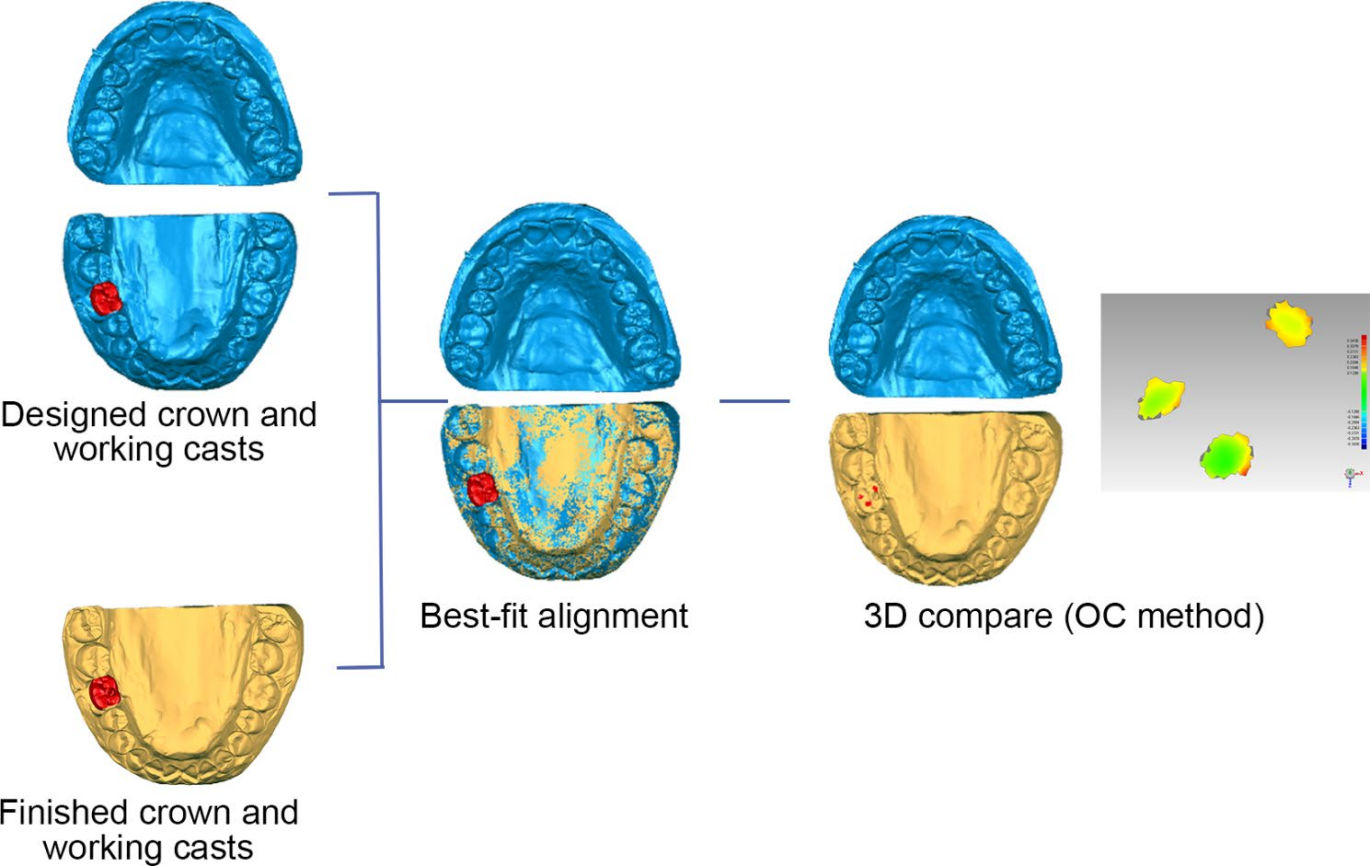
Workflow of occlusal clearance calculation using occlusal clearance (OC) method

**Fig. 4:**
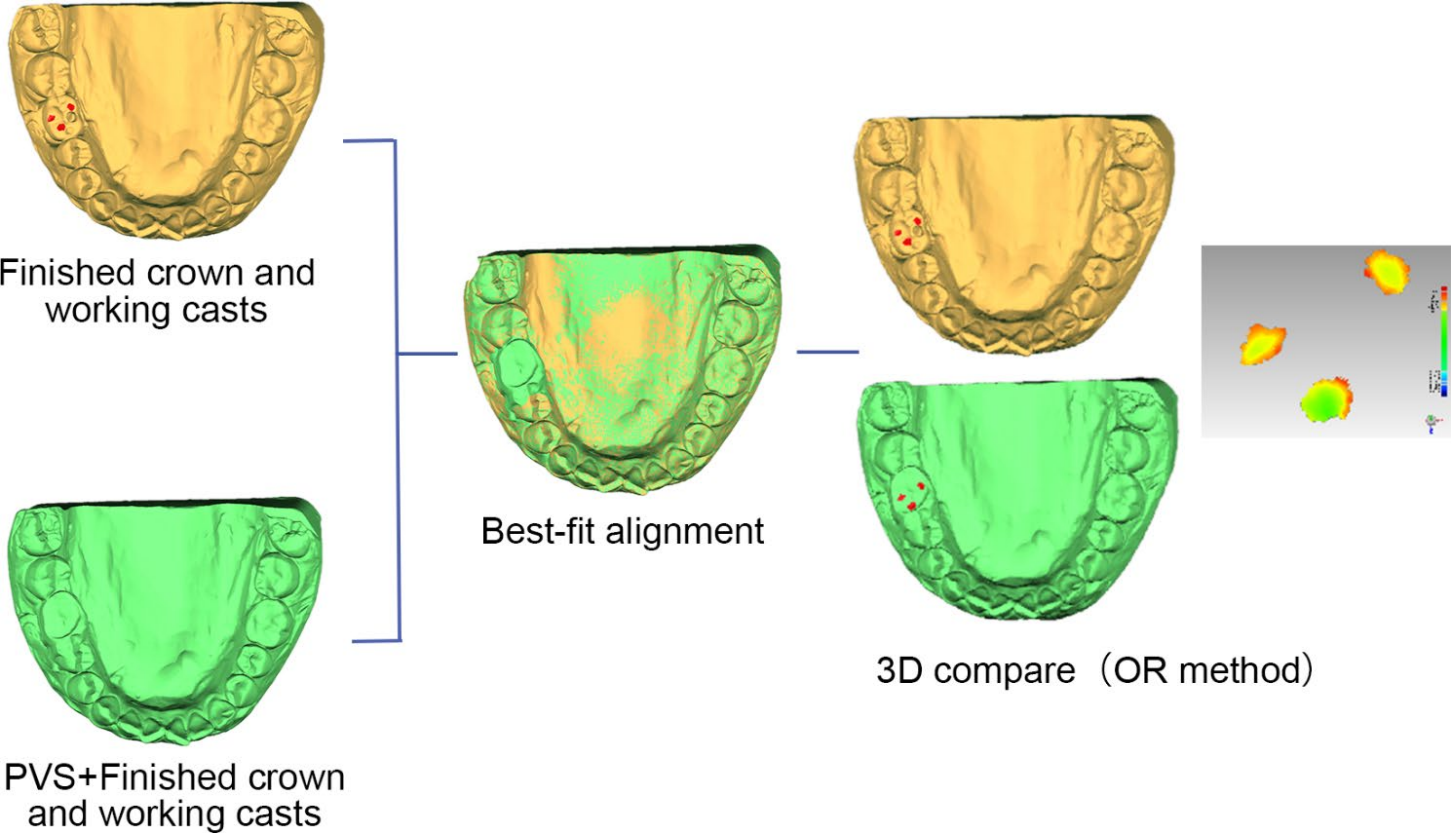
Workflow of occlusal clearance calculation using occlusal record (OR) method

Two 3D analysis methods were used to quantitatively calculated the mean and minimum occlusal clearance in the designed occlusal contact areas (hereinafter referred to as “the mean and minimum occlusal clearance”) : (i) the occlusal clearance (OC) method: calculating the distances between the designed occlusal contact area of finished crowns and the opposite teeth, the boundaries of the designed occlusal contact areas were projected from the occlusal surface of designed crown to the finished crown (Fig. 3) ; (ii) the occlusal record (OR) method: calculating the thickness of a polyvinyl siloxane (PVS, Variotime light flow; Heraeus Kulzer GmbH, Hanau, Germany) occlusal record in the designed occlusal contact areas using the following procedure. PVS was injected over the screw hole and occlusal surface of the finished crown. Subsequently, the working casts were set in MIP in the mechanical articulator, and loaded with a 3-kg weight. The articulator was carefully detached to retain the PVS occlusal record on the occlusal surface of the crown. The working casts with the PVS occlusal records were scanned, and the PVS occlusal record’s thickness in the designed occlusal contact areas was calculated by “3D compare” into the Geomagic software program (Fig. 4).

### Master cast evaluation

The implant-supported single crown was moved from the working to the master cast, and the occlusal clearance between the crown and opposite teeth was qualitatively evaluated by pulling articulating papers of 12, 30, 100, and 200 μm in succession. The occlusal contacts of the crowns were identified using a 100-µm articulating paper. The occlusal contact distributions were rated based on modified criteria (Table 1) by one researcher in random order. In this study, any working or non-working contact on the prosthesis during lateral movement is defined as lateral interference, which was examined by a 30-µm articulating paper on the mounted master casts [11]. The lateral interference ratio of the crowns was calculated. The mean and minimum occlusal clearance on the master casts were calculated using the same OC and OR methods as on the working casts. The mean occlusal clearance of the crown on the master cast was set as the primary outcome measure.

**Table 1:** Evaluation criteria of occlusal contact distribution in MIP using a 100-µm articulating paper

Score	Occlusal contact distribution
4 (Excellent)	Good occlusal contact distribution; occlusal contacts between supporting cusps and opposing fossae or ridges
3 (Good)	Individual occlusal contact points missing or are deviated, but the occlusal contacts on the main supporting cusp and crown are still functional
2 (Satisfactory)	Occlusal contacts present on other parts of the occlusal surface, but no occlusal contacts on the supporting cusps
1 (Unsatisfactory)	Distribution detrimental to crown stability or no occlusal contacts

### Statistical analysis

Data were collected and analyzed using a statistical software (IBM SPSS Statistics, version 19.0, IBM, Armonk, NY, USA). Continuous variables were assessed for normal distribution by the Shapiro-Wilk test. The 3D deviations between the designed and corresponding sintered crowns, the designed occlusal clearances, and the mean and minimum occlusal clearances were calculated as root mean square (RMS) in Geomagic software program. These results were expressed as mean ± standard deviation and compared between the two groups using the paired-samples *t*-test. The scores of occlusal contact distribution and number of occlusal contacts were expressed as median (interquartile range) and analyzed by the Mann-Whitney U test in the two design groups. The McNemar’s test compared the two groups for the articulating paper pull-out and lateral interference results. The two-tailed significance level for all statistical tests was set at *P* < 0.05.

## Results

Twenty-eight implant-supported single crowns were fabricated in this study. The mean occlusal clearance of the designed crowns in the CAD group was 92.4 ± 2.0 μm and that in the conventional group was 98.2 ± 3.3 μm (*P = 0*.183). The 3D deviation between the axial and occlusal surfaces of the designed and corresponding sintered crowns in the CAD group was 54.5 ± 12.5 μm and that in the conventional group was 44.9 ± 11.4 μm (*P* = 0.236).

**Fig. 5:**
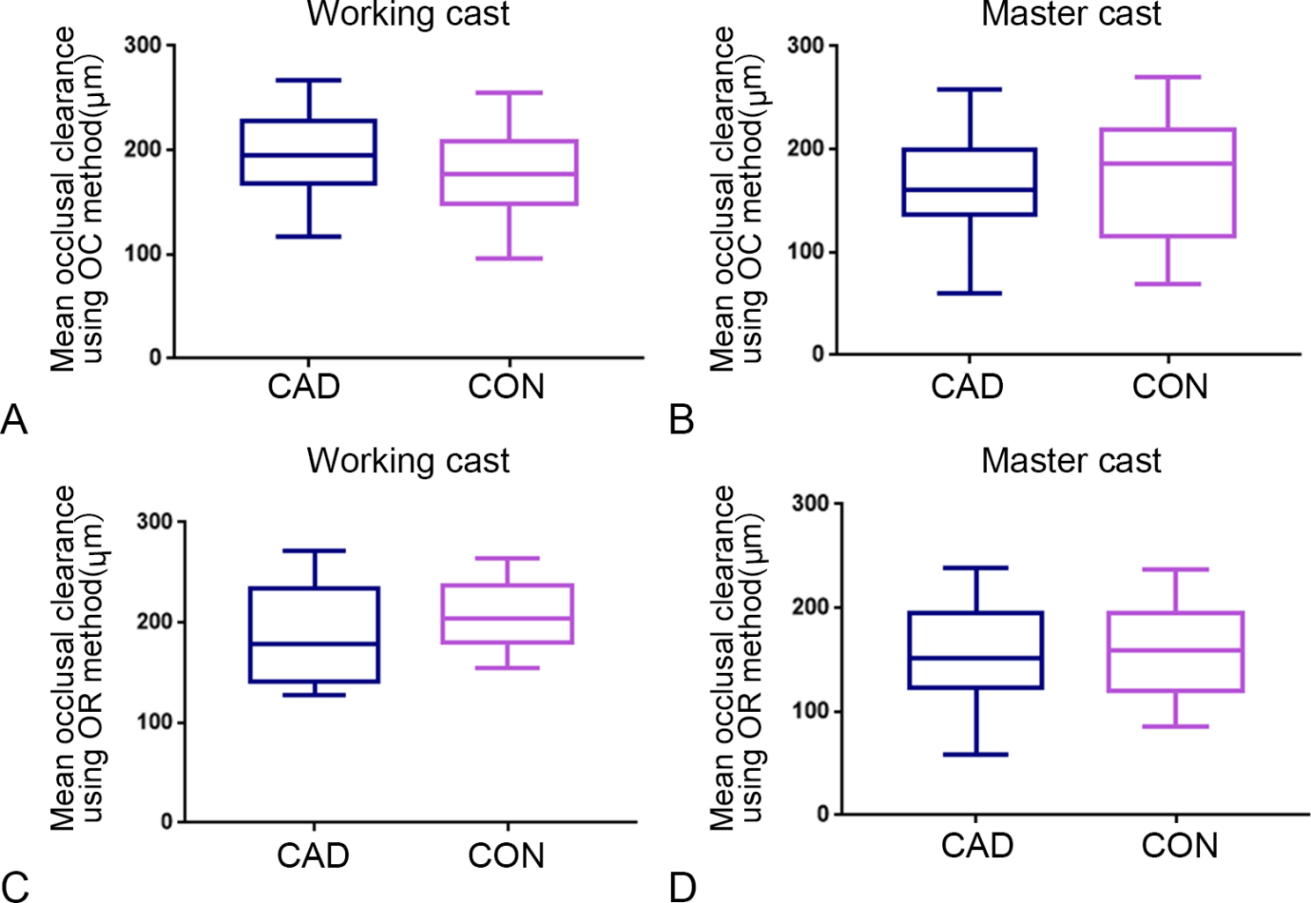
Mean occlusal clearances in designed occlusal contact areas in working and master casts: A, mean occlusal clearance in working casts using OC method; B, mean occlusal clearance in master casts using OC method; C, mean occlusal clearance in working casts using OR method; D, mean occlusal clearance in master casts using OR method (CAD, computer assisted design method; CON, conventional method; OC, occlusal clearance method; OR, occlusal record method)

The mean and minimum occlusal clearances of the two methods in the working and master casts are shown in Table 2; Fig. 5. The mean occlusal clearances on the working and master casts were significantly larger than the corresponding designed occlusal clearances (*P* < 0.001). For evaluation on the working casts by the OC method, similar mean occlusal clearances were found in the CAD and conventional groups (*P* = 0.300), while the minimum occlusal clearance of the CAD group was significantly larger than that of the conventional group (*P* = 0.043). The two design methods had similar mean and minimum occlusal clearances when assessed using the OR method (*P* = 0.224 and 0.595). When assessed by the OC and OR methods on the master casts, the two design methods had similar mean and minimum occlusal clearances (*P* > 0.05).

**Table 2:** The mean and minimum occlusal clearances in the working and master casts (Mean ± SD, µm)

Calculation method	Design method	Working cast		Master cast	
		Mean	Minimum	Mean	Minimum
OC Method	CAD	195.4 ± 43.8	139.5 ± 52.3	168.2 ± 51.2	76.6 ± 86.8
	Conventional	179.8 ± 41.8	99.8 ± 43.8	171.9 ± 64.5	71.9 ± 92.2
OR Method	CAD	189.7 ± 46.2	111.9 ± 46.4	157.3 ± 48.6	66.6 ± 60.8
	Conventional	207.0 ± 34.0	119.0 ± 40.8	155.3 ± 45.3	60.6 ± 68.9

The two design methods had similar articulating paper pull-out results in both the working and master casts (*P* = 0.721, *P* = 0.287). Table 3 shows that the two design methods had a similar number of crowns with and without lateral interference (*P* = 1.00). The number of occlusal contacts in the CAD group was 2.0 (1.0–2.3) and that in the conventional group was 1.5 (0.0–3.0) (*P* = 0.417). Table 4 shows the similar occlusal contact distribution scores in the two design methods (*P* = 0.530).

**Table 3:** Number of crowns with and without lateral interference in the two groups

conventional group	CAD group		*P*
	Lateral interference	No lateral interference	
Lateral interference	1	0	1.00
No lateral interference	1	12	

**Table 4:** Occlusal contact distribution scores in maximal intercuspal position for the two design methods

Score	Occlusal contact distributions				Sum
	1	2	3	4	
CAD group	3	3	6	2	14
conventional group	6	2	2	4	14

1, unsatisfactory; 2, acceptable; 3, good; 4, excellent.

## Discussion

This study analyzed the occlusal clearances and contacts of posterior implant-supported single crowns designed by the CAD and conventional methods. The two design methods had similar occlusal contacts and mean and minimum occlusal clearances in the master casts. Therefore, the null hypothesis that no differences would be found in occlusal contact and clearance outcomes between crowns designed with these two methods on master casts was accepted.

The occlusal clearances in the designed crowns were smaller than those in the finished crowns in both design methods on the working and master casts. Many contributing factors in the workflow and evaluation may affect the calculated occlusal clearance of the prosthesis from both groups. These include the accuracy of impression taking, model scanning, surface matching of scanbody, milling and sintering, polishing, cementing and powder spraying of prosthesis. Some of these factors can cause an increase in occlusal clearance, while others can decrease it.

For the CAD and the conventional group, the occlusal clearance of the finished crowns on the working casts appeared larger than that on the master casts. The same prosthesis had been both cemented on working and master casts and scanned, so the bonding of the prosthesis and model scanning have similar effects on the occlusal clearance for two casts. The laboratory scanner used in this study had high accuracy (± 5 μm), which acquires the image by using built-in cameras with 5 megapixels for texture mapping and features multiline technology [15, 16]. Impression taking may change the implant location on the working cast. Sang et al. found that implant analogs in gypsum casts acquired from conventional close-tray impressions were 88 ± 44 μm gingivally lower than in the reference casts [17]. This can support our results, considering that the gingivally located implant on the working cast would cause the decrease of the occlusal clearance on the master cast.

For both the CAD and conventional group, there is a larger occlusal clearance in the finished crown on the working casts than the designed one. The procedures including model scanning, surface matching of scanbody, milling and sintering, polishing, cementing could influence the measured occlusal clearance for both groups. In both groups, the designed occlusal clearance was calculated using STL file obtained by model scanning, which was similar to the scanning process for calculating occlusal clearance of finished prosthesis in the evaluation. So model scanning had little effect on the result. The surface matching of scanbody is a necessary procedure in the design of implant prostheses of two groups. The software would not enter the next step for design if the registration result was poor. It is reported that the registration accuracy would be considered poor when the registration RMS of a single object is greater than 50 μm [18]. Therefore, the surface matching of scanbody should result in a deviation less than 50 μm, which has an uncertain effect on the change of occlusal clearance. The linear shrinkage of pre-sintered zirconia blocks is reported to be about 20%. Various thicknesses and shapes of the restorations could lead to unpredictable and irregular shrinkage [19]. The milling and sintering process in this study could cause overall shrinkage of the crown with the RMS of 54.5 ± 12.5 μm in the CAD group and 44.9 ± 11.4 μm in the conventional group, resulting in larger occlusal clearance in the finished crown. Besides, polishing could also enlarge the occlusal clearance of the sintered implant prosthesis. The thickness of the layer removed by polishing was reported to be about 25 μm for the glass-ceramic crown [20]. It has been reported that cement could result in a 20-µm positional elevation for prosthesis, so cementing could decrease the occlusal clearance by at least 20 μm [21]. The thickness of the powder sprayed on the finished crown is about 20 μm, possibly decreasing the measured occlusal clearance [22]. But powdering can only decrease the occlusal clearance of the prosthesis made by the CAD method, because both the wax-up and finished prosthesis were sprayed powder before scanning in the conventional group.

According to the above analysis, milling, sintering and polishing could increase the occlusal clearance of the finished crowns, while cementing and powdering could decrease it. The result including about 100-µm larger mean occlusal clearance of the finished prosthesis on the working cast than the design is the combined effect of these factors. Based on the effect degree, the milling and sintering deviation is presumably the main reason for the increase of occlusal clearance in the finished crown on working casts.

When subjected to occlusal loading, the periodontal ligament of adjacent natural teeth could be compressed, resulting in a tighter intraoral occlusion. There is a big difference in the axial mobility between endosseous dental implants and natural teeth, 3–5 and 10–50 μm, respectively. Therefore, an appropriate occlusal clearance (10–30 μm) [9–11] of implant-support fixed prostheses should be reserved in the casts. While patients are waiting for the fabrication of restorations in clinical, their unopposed posterior teeth might continue to erupt. Guo et al. found that the unopposed posterior teeth have overerupted by about 67.9 μm during three months from implant placement to restoration insertion [23]. It can be inferred that the antagonist teeth might overerupt by about 20 μm during the 3–4 weeks of waiting for the prosthesis. The possibility of overeruption of the antagonist teeth should be considered in clinical situations. Considering the above factors, the occlusal clearance of finished crowns in the master casts should be about 30–50 μm. The calculated minimum occlusal clearances of the restorations made by the CAD and conventional groups on the master casts ranged between 60.6 and 70.6 μm. Therefore, both design methods can meet the clinical requirements of occlusal clearance design in implant-supported single crowns.

In this study, there is no difference between occlusal contact evaluation and lateral interference of the CAD group using virtual articulator and those of the conventional method using the mechanical articulator. Both groups showed few lateral interferences on the crown. It is mainly due to that transferring casts in MIP to a virtual articulator using transfer plates in a laboratory scanner was reported to be highly accurate [13]. Mehl et al. [24–26] concluded that using average-value settings in an articulator where the remaining teeth were in good condition and only single-tooth restorations or small bridges were fabricated, was equivalent to using individual settings on a semi-adjustable articulator with a facebow transfer. Zhang et al. [27] demonstrated that both the digital articulator and traditional methods using the mechanical articulator can achieve acceptable occlusal fit for single-crown restorations. In accordance to our study, the posterior implant-supported single crowns designed by the CAD and conventional methods achieved a similar occlusal fit. It is assumed that for patients with loss of natural tooth guidance and need multiple restorations or full-mouth rehabilitation, the CAD method using digital articulator may be more advantageous than the conventional method.

Although the effects of occlusal clearance design by both methods were similar, the CAD method can precisely and efficiently adjust the occlusal clearance and control the position and distribution of occlusal contacts, reducing the reliance on technicians [28]. Using the transfer plates to transfer casts in MIP to a virtual articulator can avoid the need to transport the articulators to the laboratory, thereby decreasing the errors that might occur during transit and when changing the mechanical articulator [29]. Besides, designing prostheses in virtual articulators can save costs without using combustible substrate and dental casting wax and meet environment-friendly requirements. Therefore, the CAD method is superior to the conventional method in simplifying the procedure and improving standardization of the implant prosthetic treatment. In the future, individualized occlusal contact distributions and clearances can be visualized and achieved by CAD method, promising to improve the occlusal accuracy and fit in implant-supported fixed prostheses, and conducive to lowering the risk of mechanical complications [30, 31].

This study had several limitations. First, it was an in vitro study. The dentition in the cast cannot simulate natural teeth overeruption and the periodontal ligament compression; Second, casts of only two patients with a single posterior tooth missing were selected as the master casts for this study. The differences in occlusal relationships, number and position of missing teeth, and distribution of the occlusal contacts on the dental casts should all be considered in further research. Third, a closed tray technique was used in this study, although its accuracy can meet the requirements of single implant restoration, the pick-up impression technique could be more accurate on implants position [32]. At last, the amount of deviation for every procedure should be calculated. It is very challenging to control every single step and then to understand where the difference in errors production arise. In the future, additional study that checks for every procedure the amount of deviation should be done.

## Conclusion

Within the limitations of this study, it can be concluded that posterior implant-supported single crowns designed by the CAD method were comparable to those designed by the conventional method in the occlusal clearance and occlusal evaluation outcomes. The CAD method can build a standardized process for the design and fabrication of implant-supported single crowns.

## Data Availability

The datasets used or analyzed during the current study are available from the corresponding author on reasonable request.

## Abbreviations

CAD: Computer-aided design
MIP: Maximal intercuspal position
OC: Occlusal clearance
OR: Occlusal record
STL: Standard Triangle Language
3D: Three-dimensional
PVS: Polyvinyl siloxane
RMS: Root mean square.
